# Early Warning Systems for Emerging Profiles of Antimicrobial Resistance in Italy: A National Survey

**DOI:** 10.3390/ijerph20095623

**Published:** 2023-04-24

**Authors:** Jessica Iera, Chiara Seghieri, Lara Tavoschi, Claudia Isonne, Valentina Baccolini, Daniele Petrone, Antonella Agodi, Martina Barchitta, Luca Arnoldo, Roberta Creti, Silvia Forni, Annibale Raglio, Enrico Ricchizzi, Lorenzo Bandini, Adriano Grossi, Fortunato D’Ancona

**Affiliations:** 1 Management and Health Laboratory, Institute of Management, Department EMbeDS, Sant’Anna School of Advanced Studies, 56127 Pisa, Italy; 2 Department of Public Health and Infectious Diseases, Sapienza University of Rome, 00185 Rome, Italy; 3 Department of Translational Research and New Technologies in Medicine and Surgery, University of Pisa, 56126 Pisa, Italy; 4 Department of Infectious Diseases, Istituto Superiore di Sanità, 00162 Rome, Italy; 5 Department of Statistics, Sapienza University of Rome, 00185 Rome, Italy; 6 Department of Medical and Surgical Sciences and Advanced Technologies “GF Ingrassia”, University of Catania, 95123 Catania, Italy; 7 Department of Medicine, University of Udine, 33100 Udine, Italy; 8 Accreditation and Quality Unit, Friuli Centrale Healthcare University Trust, 33100 Udine, Italy; 9 Regional Health Agency of Tuscany, 50139 Florence, Italy; 10 Division of Microbiology and Virology, ASST Papa Giovanni XXIII, 24127 Bergamo, Italy; 11 Regional Health and Social Agency, Emilia Romagna Region, 40127 Bologna, Italy

**Keywords:** antimicrobial resistance, early warning system, emerging antimicrobial resistance, surveillance

## Abstract

Antimicrobial resistance (AMR) national surveillance systems in Italy lack alert systems for timely detection of emerging profiles of AMR with potential relevance to public health. Furthermore, the existence of early warning systems (EWS) at subnational level is unclear. This study aims at mapping and characterizing EWS for microbiological threats available at regional level in Italy, focusing on emerging AMR, and at outlining potential barriers and facilitators to their development/implementation. To this end, a three-section, web-based survey was developed and administered to all Italian regional AMR representatives from June to August 2022. Twenty out of twenty-one regions and autonomous provinces (95.2%) responded to the survey. Among these, nine (45%) reported the implementation of EWS for microbiological threats at regional level, three (15%) reported that EWS are in the process of being developed, and eight (40%) reported that EWS are not currently available. EWS characteristics varied widely among the identified systems concerning both AMR profiles reported and data flow: the microorganisms most frequently included were extensively drug-resistant (XDR) *Enterobacterales*, with the lack of a dedicated regional IT platform reported in most cases. The results of this study depict a highly heterogeneous scenario and suggest that more efforts aimed at strengthening national AMR surveillance systems are needed.

## 1. Introduction

Antimicrobial resistance (AMR), which occurs when microorganisms (such as bacteria, viruses, fungi and parasites) develop the ability of being resistant to one or several antimicrobial agents, has been declared by the World Health Organization (WHO) as one of the top ten global public health threats, requiring urgent multisectoral actions [1,2]. Implications concern both clinical and economic aspects [3,4], with a global burden associated with infections due to drug-resistant microorganisms in 2019 estimated at 4.95 million deaths, of which 1.27 million were directly attributable to AMR [5]. In Europe, Italy has for years been one of the countries with the highest rates of resistance to the main classes of antibiotics used in hospitals [6,7] and one of the countries with the highest overall burden of infections by antibiotic-resistant bacteria [8]. As a result, in 2017, the Ministry of Health adopted the “National Plan Against Antimicrobial Resistance” [9].

One of the main areas of intervention identified in the plan involves surveillance, an essential tool to address policies and infection prevention and control activities whose pivotal role has been highlighted at international level [1,10]. In Italy, the national AMR surveillance is coordinated by the National Institute of Health (Istituto Superiore di Sanità, ISS) and includes several systems. Among these, the most relevant are the National Antimicrobial Resistance Sentinel Surveillance System (AR-ISS) and the Carbapenem-Resistant *Enterobacterales* bloodstream infections surveillance system (CRE). Through AR-ISS, Italy provides AMR data to the Global Antimicrobial Resistance and Use Surveillance System (GLASS) launched by the WHO and participates in the European Antimicrobial Resistance Surveillance Network (EARS-Net) coordinated by the European Centre for Disease Prevention and Control (ECDC) [11,12].

However, these AMR surveillance systems have some limitations, including the lack of an alert and early reporting system for timely detection of emerging profiles of AMR with potential relevance to public health. Furthermore, since the Italian healthcare system is a Beveridge-like model characterized by a high degree of decentralization [13] with 21 different regional health systems, the presence and organization of microbiological alert systems at subnational level are unclear. The purpose of this study was to map early warning systems (EWS) for microbiological threats at the regional level—focusing on emerging profiles of AMR—to define their main characteristics and to outline potential barriers and facilitators to their development or implementation. The findings could guide stakeholders to define a national EWS for timely reporting of microorganisms with new/unusual AMR profiles, which is one of the actions outlined in the National Plan against Antimicrobial Resistance [14].

## 2. Materials and Methods

From June to August 2022, all Italian regional AMR representatives were invited to complete a questionnaire developed through Surveymonkey^®^ (San Mateo, CA, USA), an online survey tool, which was sent by e-mail to institutional e-mail addresses. Participation was on a voluntary basis.

The questionnaire was self-administered and took approximately fifteen minutes to complete. It consisted of closed- and open-ended questions grouped into three main sections. The first section aimed at mapping EWS for microbiological threats—focusing on emerging profiles of AMR—available at the regional level in Italy. The second section explored the characteristics of existing systems (i.e., events reported and data flow). In the third section, regional AMR representatives were asked to outline barriers and facilitators to the development/implementation of EWS.

In order to achieve a multidisciplinary approach, eleven experts were identified and then invited to participate to the study, including infectious diseases experts, epidemiologists and microbiologists from the ISS (Infectious Disease Department), researchers from several regional health services (Tuscany, Emilia-Romagna, and Friuli Venezia Giulia), and public health experts from Sant’Anna School of Advanced Study—Pisa, from the University of Pisa, and from the University of Catania. The survey was developed following consensus among the identified experts and taking into consideration the framework defined by the WHO in the context of the Emerging Antimicrobial Resistance Reporting component of the Global Antimicrobial Resistance Surveillance System (GLASS-EAR) [15].

**Figure 1:**
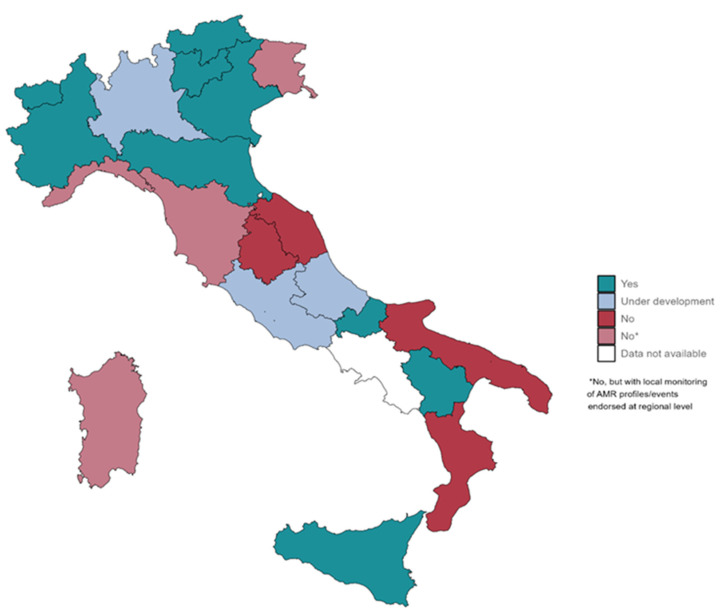
Early warning systems (EWS) available at the regional level in Italy. *Light red indicates regions where a list of AMR profiles/events requiring local monitoring and endorsed at the regional level is available.

Data were descriptively analyzed. Specifically, data were described using counts and percentages and through graphical representations (heatmaps and maps) and tables. All analyses were carried out using Excel software—version 2303, except for the map (Figure 1), which was made through RStudio 2021.09.0 software under R 4.1.2.

## 3. Results

### 3.1. Mapping Early Warning Systems at Regional Level

In total, 20 out of 21 (95.2%) regions and autonomous provinces (AA.PP.) responded to the survey (Campania Region did not participate). Among those, 45% (n = 9, i.e., Basilicata, Bolzano Autonomous Province, Emilia-Romagna, Molise, Piedmont, Trento Autonomous Province, Veneto, Sicily and Valle D’Aosta) reported the implementation of EWS for microbiological threats; 15% (n = 3, i.e., Abruzzo, Lazio and Lombardy) reported that EWS are under development; 40% (n = 8, i.e., Calabria, Friuli Venezia Giulia, Liguria, Marche, Puglia, Sardinia, Tuscany and Umbria) reported that EWS at the regional level are currently not available. Among the eight regions without a structured alert system, 50% (n = 4, i.e., Friuli Venezia Giulia, Liguria, Sardinia and Tuscany) reported the presence of a list of AMR profiles/events requiring local monitoring and endorsed at the regional level (Figure 1).

Among the three regions where EWS are under development and the eight regions without a regional alert system, two out three and seven out of eight reported, respectively, the availability of EWS for microbiological threats—including emerging AMR—at the local level, where health services are delivered by Local Health Authorities (“Aziende Sanitarie Locali”).

### 3.2. Key Characteristics of Existing Regional Early Warning Systems

**Figure 2:**
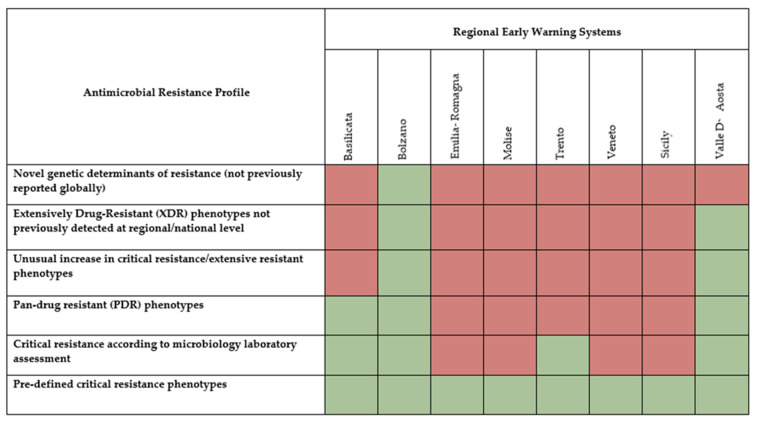
Antimicrobial resistance profiles included in existing Italian regional early warning systems. Eight out of nine regions/AA.PP. provided information. Green, AMR profile included; red, AMR profile not included.

Among the nine available regional EWS, eight provided information concerning AMR profiles that are expected to be reported: one system included novel genetic determinants of resistance (not previously reported globally), two systems included extensively drug-resistant (XDR) phenotypes not previously detected at regional/national level, two systems included an unusual increase in critical resistance/extensive resistant phenotypes, three systems included pan-drug resistant (PDR) phenotypes, four systems included critical resistance according to microbiology laboratory assessment, and all systems included pre-defined critical resistance phenotypes (Figure 2).

#### 3.2.1. Microorganisms’ Critical Resistance Phenotypes

**Figure 3:**
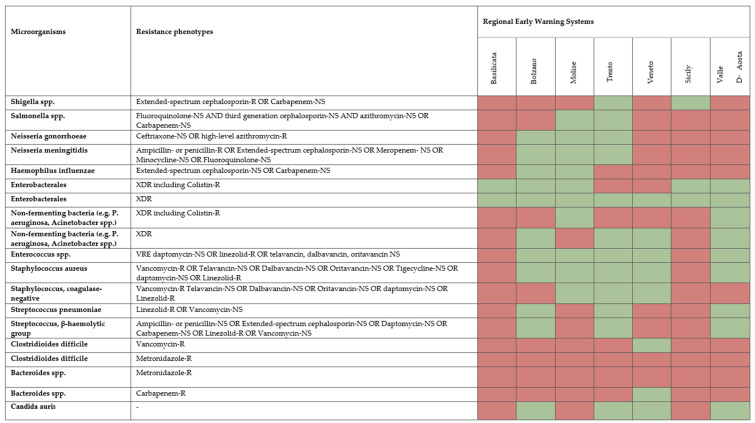
Critical resistance phenotypes (R, resistant; NS, non-susceptible) reported at the regional level in Italy. Seven out of eight regions/AA.PP. provided information. Green, critical resistance phenotypes reported; red, critical resistance phenotypes not reported.

Seven out of eight EWS that included pre-defined critical resistance phenotypes provided further details on critical resistance phenotypes that are expected to be reported (Figure 3).

The most frequent profiles were the following: XDR *Enterobacterales* (seven out of seven); XDR including Colistin-resistant *Enterobacterales* (five out of seven); Vancomycin-resistant Daptomycin-nonsusceptible (NS) or Linezolid-resistant or Telavancin-, Dalbavancin-, Oritavancin-NS *Enterococcus spp.* (five out of seven); and Vancomycin-resistant or Telavancin-NS or Dalbavancin-NS or Oritavancin-NS or Tigecycline-NS or Daptomycin-NS or Linezolid-resistant *Staphylococcus aureus* (five out of seven). None of the existing EWS included Metronidazole-resistant *Clostridioides difficile* and Metronidazole-resistant *Bacteroides* spp.

#### 3.2.2. Specimen Collection and Reporting

In all available regional EWS, the setting where specimens are collected was hospital-based; in five out of nine (Bolzano A.P., Emilia-Romagna, Piedmont, Trento A.P. and Valle D’Aosta), the setting included long-term care facilities, whereas in four regions/AA.PP. (Bolzano A.P., Emilia-Romagna, Piedmont and Valle D’Aosta), the setting included the community as well.

In two out of nine available regional EWS (Molise and Trento A.P.), reporting came from reference laboratories; in four out of nine (Bolzano A.P., Molise, Piedmont and Trento A.P.), reporting involved any microbiology laboratory, and isolates were sent to a reference laboratory when deemed appropriate; in three out of nine (Basilicata, Piedmont and Sicily), reporting involved any microbiology laboratory (systematic transfer of specimens to a reference laboratory is not planned); in five out of nine (Basilicata, Emilia-Romagna, Piedmont, Veneto and Sicily), reporting came from health directorates; in two out of nine (Piedmont and Trento A.P.), reporting came from public health departments.

The reporting procedure involved the use of a web form/software in six out of nine available regional EWS (Bolzano A.P., Emilia-Romagna, Piedmont, Trento A.P., Sicily and Valle D’Aosta). The procedure was paper-based in three out of nine EWS (Basilicata, Piedmont and Trento A.P.), and other methods (such as phone calls, e-mails, etc.) were included in six out of nine EWS (Bolzano A.P., Molise, Piedmont, Trento A.P., Valle D’Aosta and Veneto).

Clinical microbiologists were primarily in charge of reporting in seven out of nine available regional EWS (all systems except Emilia-Romagna and Veneto Regional EWS, where reporting involved risk managers). Reporting was provided to health directorates in most cases (seven out of nine: all available systems except for Emilia-Romagna regional EWS, where reporting was provided to prevention departments and public health regional authorities, and Veneto EWS, where reporting was provided to the regional Clinical Risk Department).

Reporting times varied widely: of the nine regional EWS, real-time reporting was provided in two EWS (Piedmont and Valle D’Aosta); in two EWS (Bolzano A.P. and Emilia-Romagna), reporting was provided within 24 h and in one (Sicily) within 48 h, in one (Veneto) on a monthly basis, in one (Basilicata) within 72 h, and in two EWS (Molise and Trento A.P.) at the time of results validation.

A dedicated regional IT platform for structured data sharing between local and regional levels was in place in three out of nine regional EWS (Emilia-Romagna, Trento A.P., and Sicily) and not available in the six remaining systems.

Pre-defined reporting was provided by five out of nine EWS (Emilia-Romagna, Trento A.P., Veneto, Bolzano A.P. and Sicily), while pre-defined reporting was not included in four EWS (Basilicata, Molise, Piedmont and Valle D’Aosta).

#### 3.2.3. Post-Reporting Actions

Actions taken following the reporting were outlined in six out of nine available EWS (Molise, Piedmont, Trento, Bolzano, Sicily and Valle D’Aosta) and included, in all instances, the implementation of infection and control measures at local level.

Performance measurement and evaluation of available EWS were not implemented in eight out of nine EWS; in one out of nine (Trento A.P.), EWS performance measurement and evaluation were implemented, but no further details are available.

Data quality monitoring was provided in three out of nine (Molise, Piedmont and Trento) EWS and not included in the six remaining systems (Basilicata, Emilia-Romagna, Veneto, Bolzano, Sicily and Valle D’Aosta).

#### 3.2.4. Availability of Formalized Laboratories Network

Among the nine regions/AA.PP. that implemented EWS, seven provided information concerning the availability of a formalized network of reference laboratories (RL) for confirmation of unusual antimicrobial resistance. Among these, in four out of seven (Molise, Trento, Bolzano and Sicily), a network of reference laboratories was available; in two out of seven (Emilia-Romagna and Valle D’Aosta), a network of RL was not available; in one out of seven (Piedmont), the network was in the process of being developed.

A summary of the results outlined above is provided in Table 1.

### 3.3. Barriers and Facilitators

Among the 20 respondents, 17 provided information concerning barriers to the development and/or the implementation of regional EWS, while 14 provided information concerning facilitators.

The main barriers were identified as the lack of appropriate software, privacy issues, and poor cooperation among microbiology laboratories.

The main facilitators were outlined as proper data collection and appropriate technology availability, the presence of a network of microbiology laboratories, and the clear definition of responsibilities in the context of the system.

## 4. Discussion

Emerging antimicrobial resistance (AMR) may represent a concerning international public health risk [15]. In October 2015, during the first meeting of the GLASS Platform [16] launched by the WHO [17], participants agreed that there was an urgent need to develop a system for early detection and reporting that would highlight emerging AMR mechanisms and map their global spread.

As a result, the GLASS-EAR component was developed, providing a tool embedded in the GLASS IT platform where experts can share information concerning emerging AMR events that may have an impact on surveillance and control practices [15,18].

However, the availability of EWS for monitoring emerging AMR at the international level has not yet been deeply investigated, and the publicly available information on existing alert systems is not broadly detailed, particularly concerning methodologic aspects. Focusing on newly launched antibiotics within the healthcare setting, the requirements needed to develop a standardized resistance monitoring system were explored in a recently published study. The authors deemed as particularly relevant the implementation of an early warning surveillance as soon as a newly introduced antimicrobial agent is used locally, taking into consideration that current AMR surveillance systems mainly focus on resistance to older antimicrobial agents, and the measurement of resistance to newly launched drugs such as those considered “last resort” options seems to not yet be broadly implemented in the context of the public sector [19].

In Italy, data on the consumption of “last resort” antibiotics, according to the AWaRe classification developed by the WHO [20], are available. [21] However, national surveillance systems currently lack EWS for emerging AMR. In addition, findings from the subnational level collected through this paper, which presents the results of the first survey conducted at the regional level in Italy on EWS for microbiological threats that focus on microorganisms with new/unusual AMR profiles, depicted a highly heterogeneous scenario.

Less than half of Italian regions have implemented alert systems at the regional level, most of which are available in northern Italy, reflecting the imbalance between the north and south of the country [22]. Furthermore, these systems are mainly designed for microbiological alerts management at the local/regional level, while data sharing at the national level is not yet implemented, in contrast to other surveillance systems available in other countries, which seem to provide useful information on emerging AMR with national coverage. Among European countries, for example, Sweden is currently using several systems for national coverage of AMR surveillance, including Svebar, a system where all culture findings from the country’s laboratories are automatically transferred on a daily basis, allowing an early alert on findings of serious antibiotics resistance [23]. Among the countries of the WHO Western Pacific Region, Australia ensures the currency of data collections by providing a systematic and timely identification of the emergence of critical antimicrobial resistances through the Antimicrobial Use and Resistance in Australia (AURA) surveillance system [24].

Among existing Italian regional alert systems, our study highlighted the presence of differences involving several aspects. Most of the AMR profiles investigated were included only in two regional EWS, with novel genetic determinants of resistance reported only in one system and pre-defined critical resistance phenotypes included in all existing EWS. However, none of the systems were homogeneous with the others, especially with regard to data flow. In particular, timing for reporting was defined as real-time or on a daily basis only in a few regional EWS. These findings could be explained considering that regional organizational models can be quite different, with data collection involving from one to multiple microbiology laboratories, and are consistent with a recently performed analysis of surveillance for control of antimicrobial resistance in Europe, which found that only 3% of the surveillance systems in Europe provided real-time access to AMR data [25].

Of interest, moreover, the lack of a dedicated regional IT platform in most cases and the availability of a network of reference laboratories in four out of nine regional EWS are findings that are consistent with some of the reported barriers to the development/implementation of alert systems.

These aspects need to be addressed in the process of strengthening national AMR surveillance systems, including the need for an adequate reference laboratories network at the national level and the need to achieve technological improvements. The scenario also highlights the importance of identifying examples of good practice at the subnational level and of achieving cooperation and coordination between the regional and the central levels, considering that AMR has been outlined as a serious national and regional challenge requiring a comprehensive and coordinated response [16]. These considerations are in line with the project “good practices for the surveillance and control of antimicrobial resistance” funded by the Italian National Centre for Disease Prevention and Control (CCM). In this context, during a meeting attended by several representatives from Italian regions, the participants agreed on the need for a national surveillance system for newly emerged resistance that should be web-based and that accreditation for participating laboratories should be provided [26].

The identification of a clear strategy for delivering sustainable surveillance through existing capacity and potential for innovation was outlined as an opportunity for addressing challenges as part of effective and sustainable efforts to manage the AMR threat in a recently performed analysis of existing national action plans for AMR [27].

Several initiatives are currently ongoing in Europe in order to strengthen national surveillance and outbreak investigation capacities and improve molecular surveillance data availability and quality as well at the European level. [28]

Italy is among the participating countries of the EURGen-RefLabCap project (“provision of EU networking and support for public health reference laboratory functions for antimicrobial resistance in priority healthcare associated infections”) coordinated by the Technical University of Denmark (DTU Food, Denmark) and the Statens Serum Institut (SSI, Denmark) in close cooperation with the European Centre of Disease Prevention and Control (ECDC). The project aims to support national references laboratories (NRLs) in improving their capacities in detection and phenotypic and genotypic characterization of Carbapenem- and/or Colistin-Resistant *Enterobacterales* (CCRE) and additional healthcare-associated pathogens of public health relevance [29].

Furthermore, according to the most recent report on antibiotics use in Italy, a proposal for the reorganization and strengthening of microbiology laboratories networks will be provided by the National Agency for Regional Health Services (AGENAS), aiming at supporting the National Plan Against Antimicrobial Resistance (PNCAR) [21].

This study has some limitations. Firstly, we provided a general picture at the regional level, but we did not conduct an in-depth analysis concerning EWS available at the local level, and we did not explore the coordination between human, veterinary, and food surveillance systems, which is essential according to the “one health” approach [30]. Secondly, we did not investigate microbiology laboratories’ capacities and capabilities, which is a relevant issue considering that microbiology laboratories provide a first line of defense against health threats from communicable diseases, including antimicrobial resistance [31]. Thirdly, the reporting of molecular epidemiology data was not widely explored in this paper. Several aspects need further investigation, including aspects concerning phenotypic and genetic characterization of isolates, particularly the methods used by microbiology laboratories to perform antimicrobial susceptibility testing, the EUCAST (the European Committee on Antimicrobial Susceptibility Testing) expert rules applied and the methodologies addressing AMR molecular aspects used, considering the relevance of understanding how bacteria and genetic elements spread, in order to establish whether AMR trends are caused by resistant strain spread or by resistance determinant transfer among different strains and species [32]. The inadequate standardization of collected data and methods of microbiological testing (including susceptibility testing) and lack of routine inclusion of genetic typing are considered relevant limitations of AMR surveillance in Europe [25]. In Italy, the development and implementation of a national EWS could provide the opportunity to improve microbiology laboratories’ capacities and the standardization of laboratory information systems (LIS).

In conclusion, to the best of our knowledge, this is the first study that explored the availability and the characteristics of early warning systems for emerging profiles of AMR at the regional level in Italy, providing relevant data to regional and national authorities for the achievement of one of the goals included in the National Plan Against Antimicrobial Resistance.

## 5. Conclusions

The results of this study suggest that more efforts aimed at strengthening AMR surveillance systems currently in place in Italy are needed, including the identification of the key elements required to develop an effective national early warning system with a bidirectional data flow. Technological issues such as the availability and standardization of dedicated laboratory information management software, which could allow real-time data collection and reporting with regional coverage, need to be addressed. Furthermore, a multidisciplinary and cross-sectoral approach should be considered, establishing common objectives and methods in order to provide the integration of animal, food, and human data and timely data sharing at the national and subnational levels, preventing the spread of microorganisms that may threaten public health also at the international level.

## Figures and Tables

**Table 1:** Main characteristics of existing early warning systems in Italy by region.

	Specimen Collection Setting	Reporting	Reporting Origin	Reporting Target	Timing	Regional IT Platform	Pre-Defined Reporting	Actions	EWS Performance Measurement	Data Quality Monitoring	Formalized RL Network
Basilicata	HB	PB	Clinical microbiologist	Health directorate	72 h	No	No	NA	No	No	NA
Bolzano A.P.	HB LTCF CB	WB Other methods	Clinical microbiologist	Health directorate	24 h	No	Yes	IPC measures	No	No	Yes
Emilia-Romagna	HB LTCF CB	WB	Risk manager	Prevention department	24 h	Yes	Yes	NA	No	No	No
Molise	HB	Other methods	Clinical microbiologist	Health directorate	Results validation	No	No	IPC measures	No	Yes	Yes
Piedmont	HB LTCF CB	WB PB Other methods	Clinical microbiologist	Health directorate	Real-time	No	No	IPC measures	No	Yes	In progress
Sicily	HB	WB	Clinical microbiologist	Health directorate	48 h	Yes	Yes	IPC measures	No	No	Yes
Trento A.P.	HB LTCF	WB PB Other methods	Clinical microbiologist	Health directorate	Results validation	Yes	Yes	IPC measures	Yes	Yes	Yes
Valle D’Aosta	HB LTCF CB	WB Other methods	Clinical microbiologist	Health directorate	Real-time	No	No	IPC measures	No	No	No
Veneto	HB	Other methods	Risk manager	Clinical risk department	Monthly	No	Yes	NA	No	No	NA

HB, hospital-based; LTCF, long-term facilities; CB, community-based; WB, web-based; PB, paper-based; EWS, early warning system; NA, information not available; RL, reference laboratories.

## Data Availability

The data presented in this study are available on request from the corresponding author.
